# Supplementary material for: Chromatin accessibility and pioneer factor FOXA1 restrict glucocorticoid receptor action in prostate cancer
Source: Nucleic Acids Res. 2023 Nov 28;52(2):625–42. doi: 10.1093/nar/gkad1126 (PMC10810216; doi:10.1093/nar/gkad1126)
Supplement: gkad1126_Supplemental_Files [file gkad1126_supplemental_files.zip › Rev_GR-ENZ_supplementary-material_NAR.pdf]

Supplementary materials for

Chromatin Accessibility and Pioneer Factor FOXA1 Restrict Glucocorticoid Receptor Action in Prostate Cancer

Laura Helminen, Jasmin Huttunen, Melina Tulonen, Niina Aaltonen, Einari A. Niskanen, Jorma J. Palvimo, Ville Paakinaho

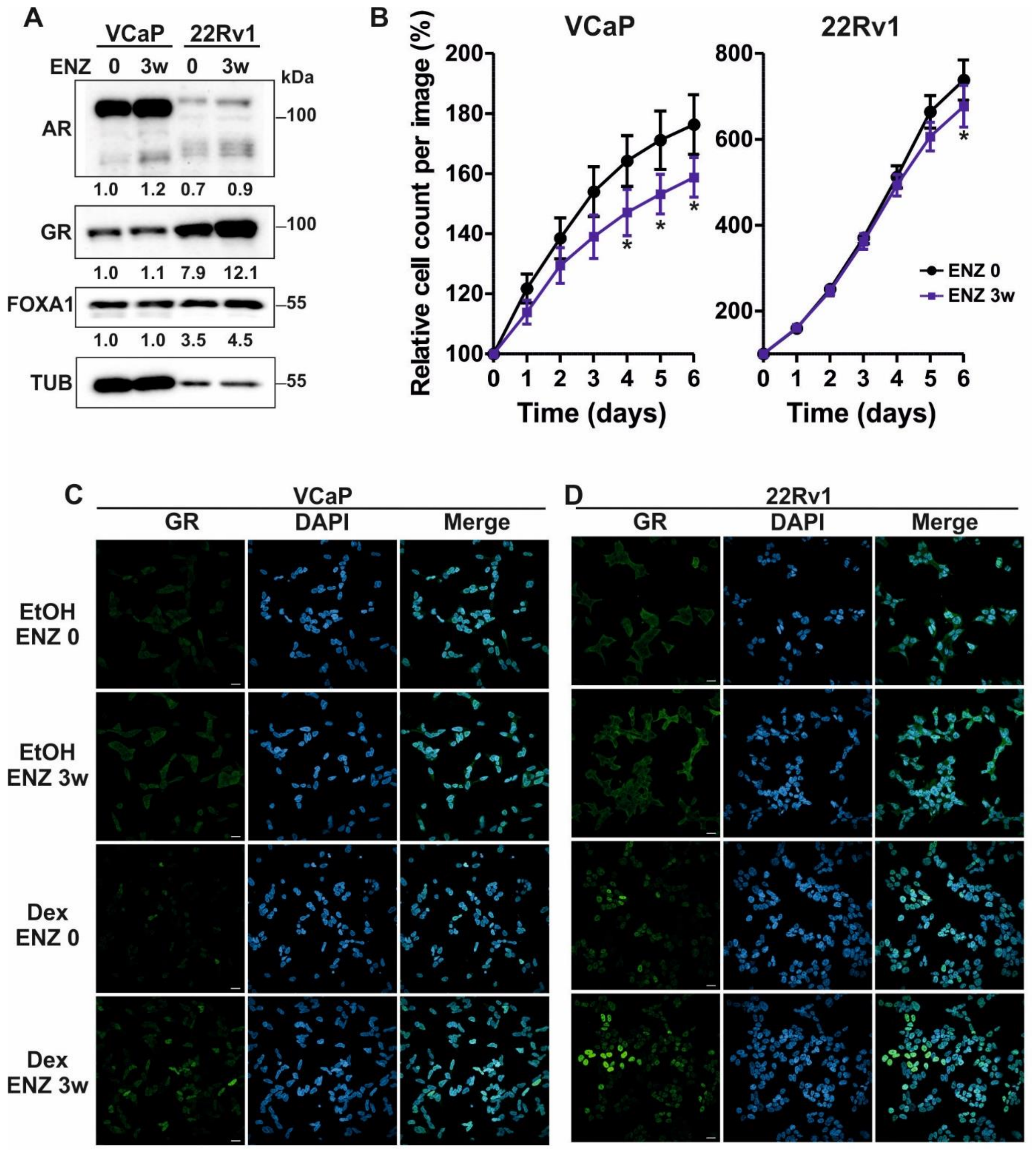

Supplementary Figure S1. The effects of enzalutamide on the protein levels of GR, cell proliferation, and GR's cellular localization. (A) Immunoblotting of AR, GR, FOXA1, and tubulin (TUB) protein levels in VCaP and 22Rv1 ENZ 0 and ENZ 3w cells. Quantification of AR, GR, and FOXA1 levels to TUB is shown below the immunoblot. (B) Relative cell proliferation of VCaP and 22Rv1 ENZ 0 (black) and ENZ 3w (blue) cells. Data is normalized to day 0 and represents the mean ±SD of 8 images. Statistical significance calculated with Two-way ANOVA with Bonferroni *post hoc* test. *, $p<0.05$. (C-D) GR immunofluorescence (IF) images from VCaP (C) and 22Rv1 (D) cells. The cells were treated with vehicle (EtOH), ENZ 3w, Dex, or ENZ 3w and Dex. DAPI was used as a marker for nuclei. Scale bar, 20 µm.

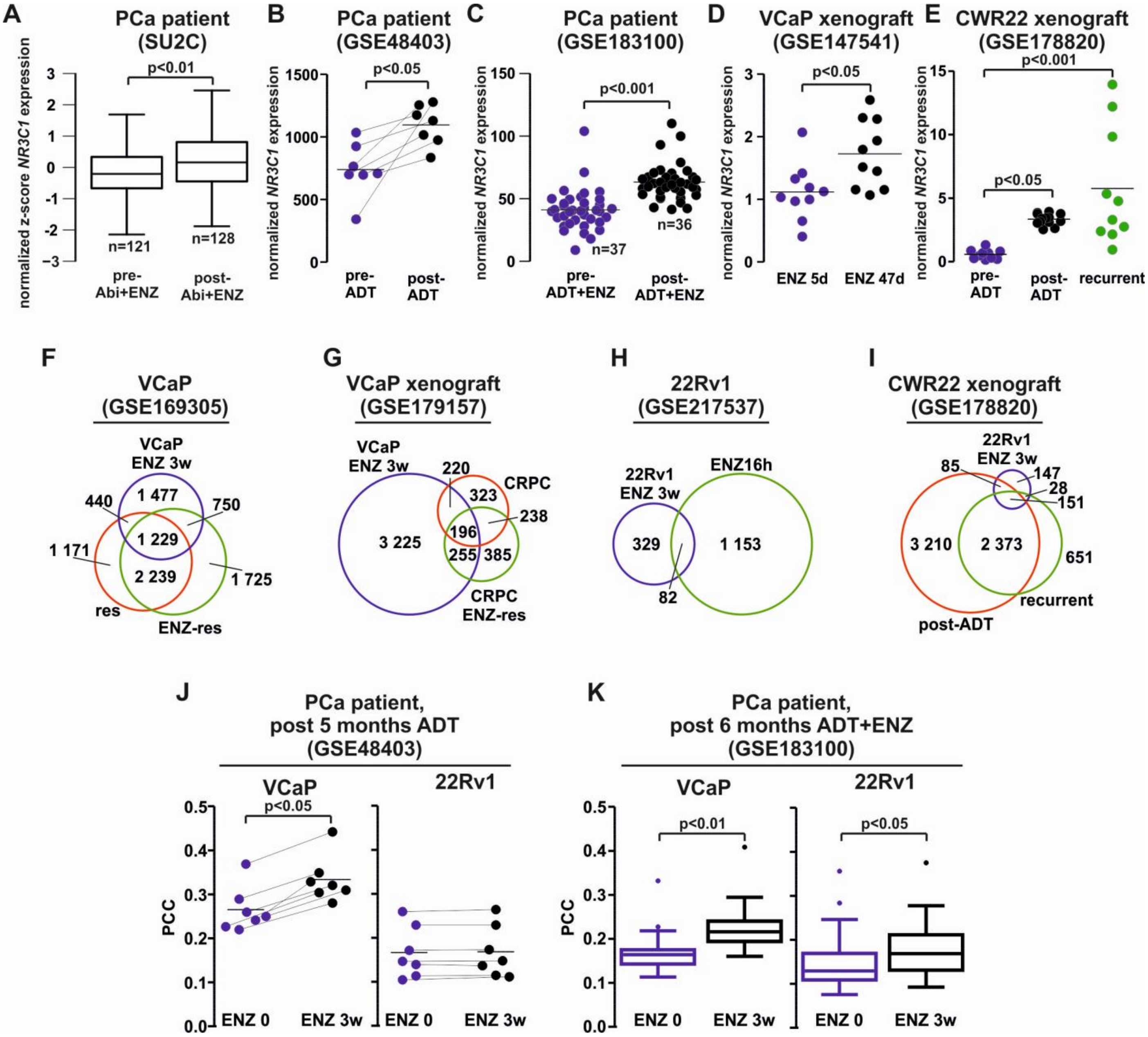

Supplementary Figure S2. Comparison of ENZ-regulated genes with public data sets. (A-E) Transcript levels of *NR3C1* by RNA-seq from (A) PCa patients pre- or post-exposure with abiraterone (Abi) and/or enzalutamide (ENZ), (B) matched PCa patients pre- or post-exposure with androgen deprivation therapy (ADT), (C) PCa patients pre- or post-exposure with ADT and ENZ, (D) VCaP xenograft after 5 (5d) or 47 days (47d) exposure to ENZ, and (E) CWR22 xenograft pre- or post-exposure with ADT and recurrent samples. (F) Comparison of differentially expressed genes between ENZ 3w (blue color), androgen-independent (res; red color) and enzalutamide-resistant (ENZ-res; green color) VCaP cells. (G) Comparison of differentially expressed genes between ENZ 3w VCaP cells (blue color), CRPC (red color) and enzalutamide-resistant CRPC (ENZ-res; green color) VCaP xenografts. (H) Comparison of differentially expressed genes between ENZ 3w (blue color) and ENZ 16h (green color) 22Rv1 cells. (I) Comparison of differentially expressed genes between ENZ 3w 22Rv1 cells (blue color), post-ADT (red color) and recurrent (green color) CWR22 xenografts. Venn diagrams depict the overlap and number of regulated target genes. (J-K) Pearson correlation coefficiency (PCC) of ENZ-regulated genes in VCaP (left) or 22Rv1 (right) cells between ENZ 0 or ENZ 3w and (J) matched PCa patients post 5-month exposure with ADT, or (K) PCa patients post 6-month exposure with ADT and ENZ. Data accession number is indicated for each dataset. Statistical significance calculated with unpaired t-test (2 conditions) or using One-way ANOVA with Bonferroni *post hoc* test (3 conditions).

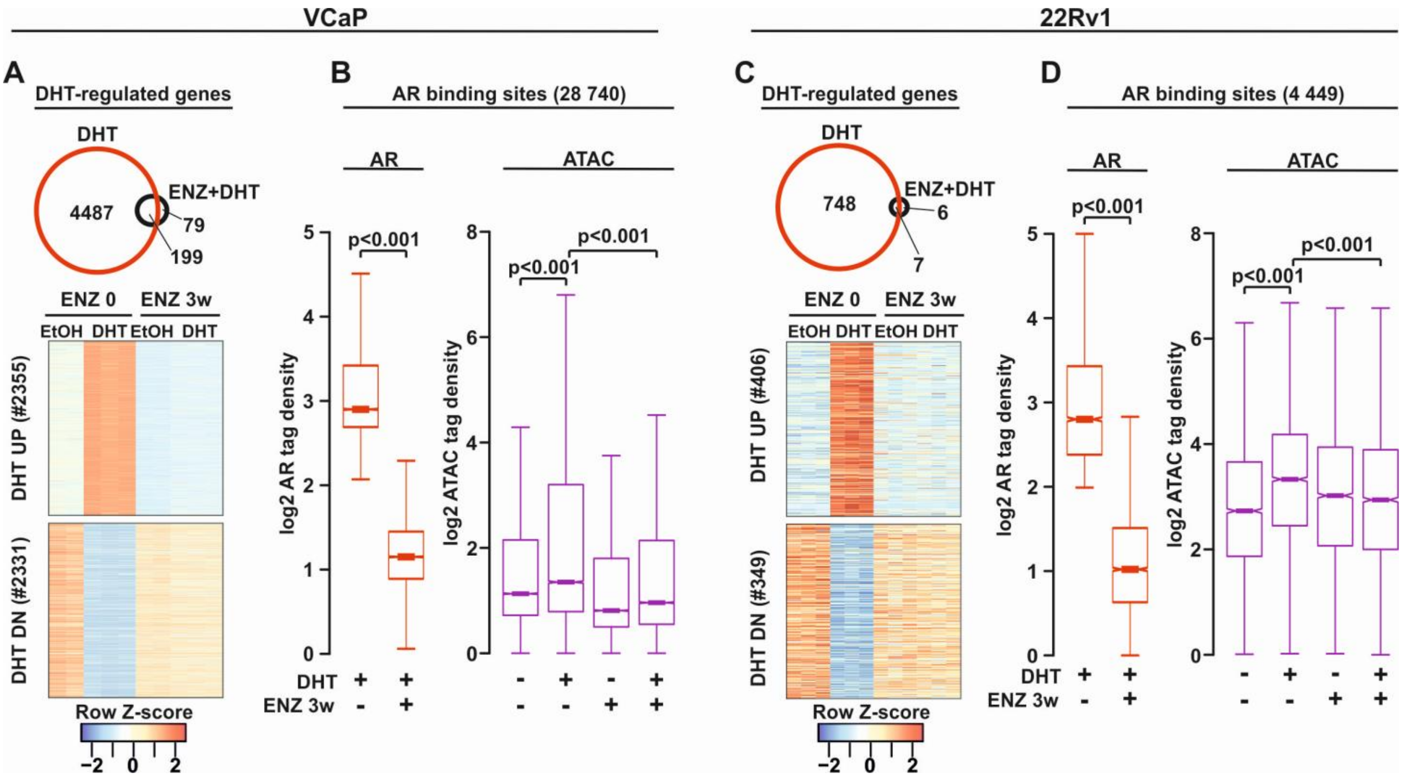

Supplementary Figure S3. Repression of AR signaling by enzalutamide. (A-D) Inhibition of AR signaling by ENZ 3w in VCaP (A-B) and 22Rv1 (C-D) cells at the level of AR target gene regulation by RNA-seq (A, C), and AR chromatin binding by ChIP-seq and AR-induced chromatin accessibility by ATAC-seq (B, D). Venn diagrams depict the overlap and number of regulated target genes, and heatmaps are displayed as row Z-score. Box plots represent the normalized log2 tag density of AR ChIP-seq and ATAC-seq at AR binding sites. The number of AR binding sites is indicated for each cell lines. All box plots are normalized to a total of 10 million reads. Statistical significance calculated with unpaired t-test (2 conditions) or using One-way ANOVA with Bonferroni *post hoc* test (3 conditions).

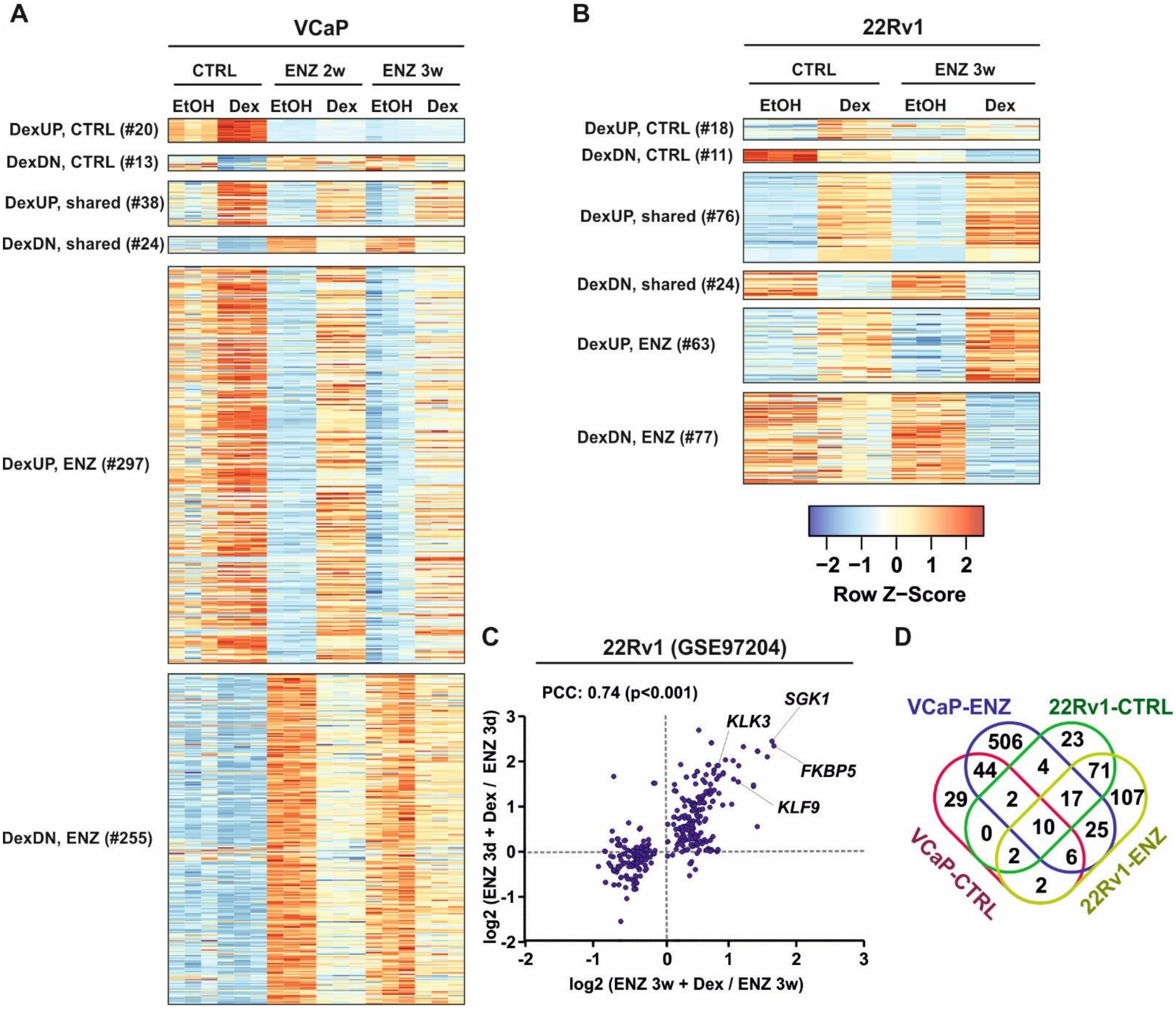

Supplementary Figure S4. RNA-seq of Dex-regulated target genes in VCaP and 22Rv1 cells. A-B) Heatmaps of Dex-regulated genes in (A) VCaP and (B) 22Rv1 cells before and after indicated ENZ treatment. Gene clusters separated based on target gene regulation only at before ENZ (CTRL), in both before and after ENZ (shared), and only after ENZ treatment. (C) Scatter plot of RNA-seq log2 TPM values of ENZ 3 weeks (3w) + Dex / ENZ 3w (x-axis) and ENZ 3 days (3d) + Dex / ENZ 3d (y-axis). Pearson correlation coefficiency (PCC) and statistical significance indicated above the plot. GR-regulated target genes *KLK3*, *KLF9*, *FKBP5* and *SGK1* indicated in the graph. (D) Venn diagrams overlap of Dex-regulated genes between VCaP and 22Rv1 cells before and after ENZ treatment.

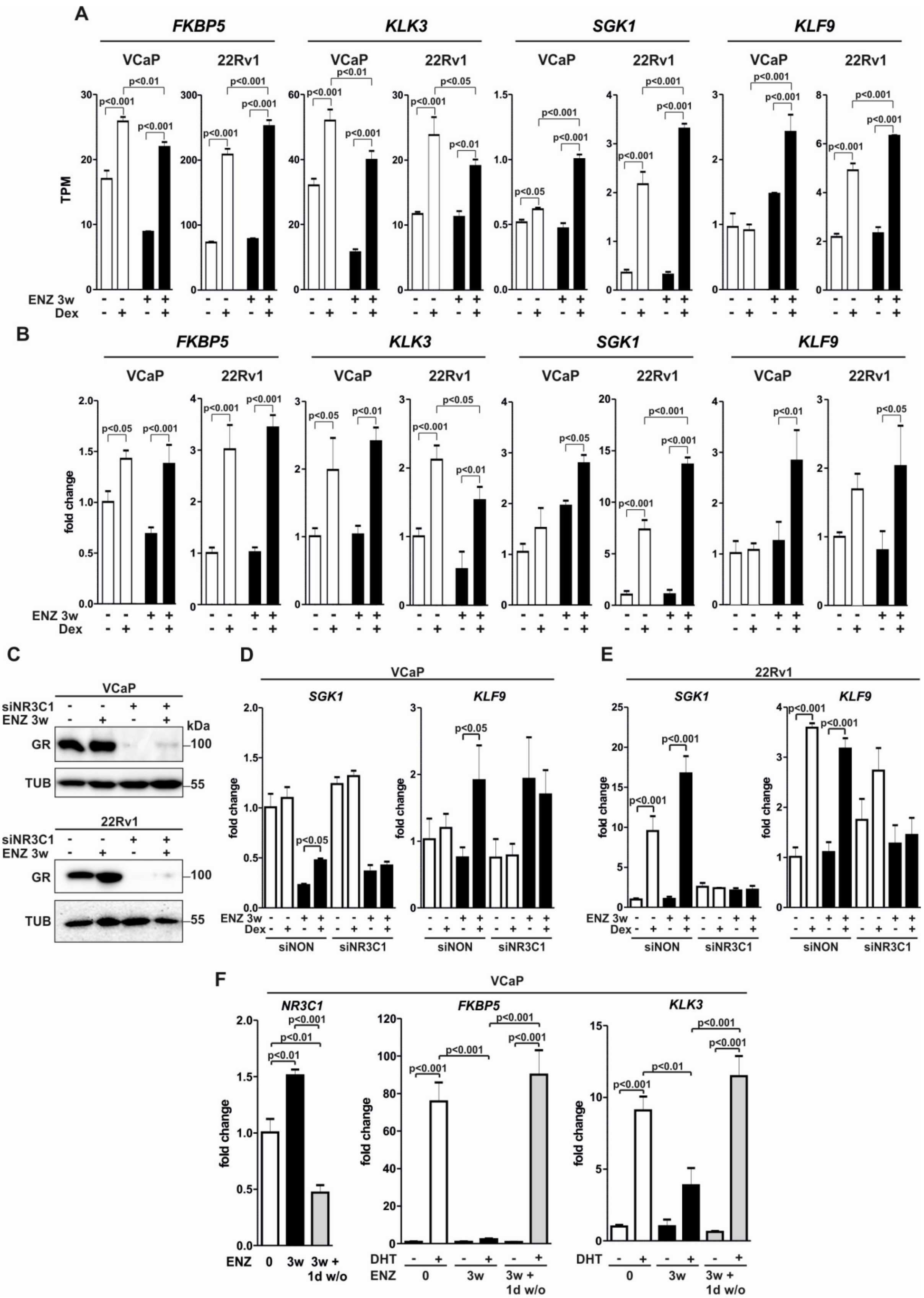

Supplementary Figure S5. Validation of Dex-regulated genes in enzalutamide treated VCaP and 22Rv1 cells. (A) Bar graphs depict transcript per million (TPM) of *FKBP5*, *KLK3*, *SGK1*, *KLF9* in VCaP and 22Rv1 ENZ 0 and ENZ 3w cells in the presence or absence of Dex from RNA-seq data. (B) Bar graphs depict GR target gene analysis of *FKBP5*, *KLK3*, *SGK1*, *KLF9* in VCaP and 22Rv1 ENZ 0 and ENZ 3w cells in the presence or absence of Dex. (C) Immunoblotting of GR and TUB protein levels in VCaP (upper) and 22Rv1 (lower) ENZ 0 and ENZ 3w cells treated with siNON or siNR3C1. (D-E) Bar graphs depict GR target gene analysis of *SGK1* and *KLF9* in VCaP (D) and 22Rv1 (E) ENZ 0 and ENZ 3w cells treated with siNON or siNR3C1 in the presence or absence of Dex. (F) Bar graphs depict target gene analysis of NR3C1, *FKBP5* and *KLK3* in VCaP ENZ 0 cells, ENZ 3w cells, or cells treated with ENZ 3w followed by 1 day without ENZ (1d w/o) in the presence or absence of DHT. Bars represent mean ±SD, n=3. Statistical significance calculated using One-way ANOVA with Bonferroni *post hoc* test.

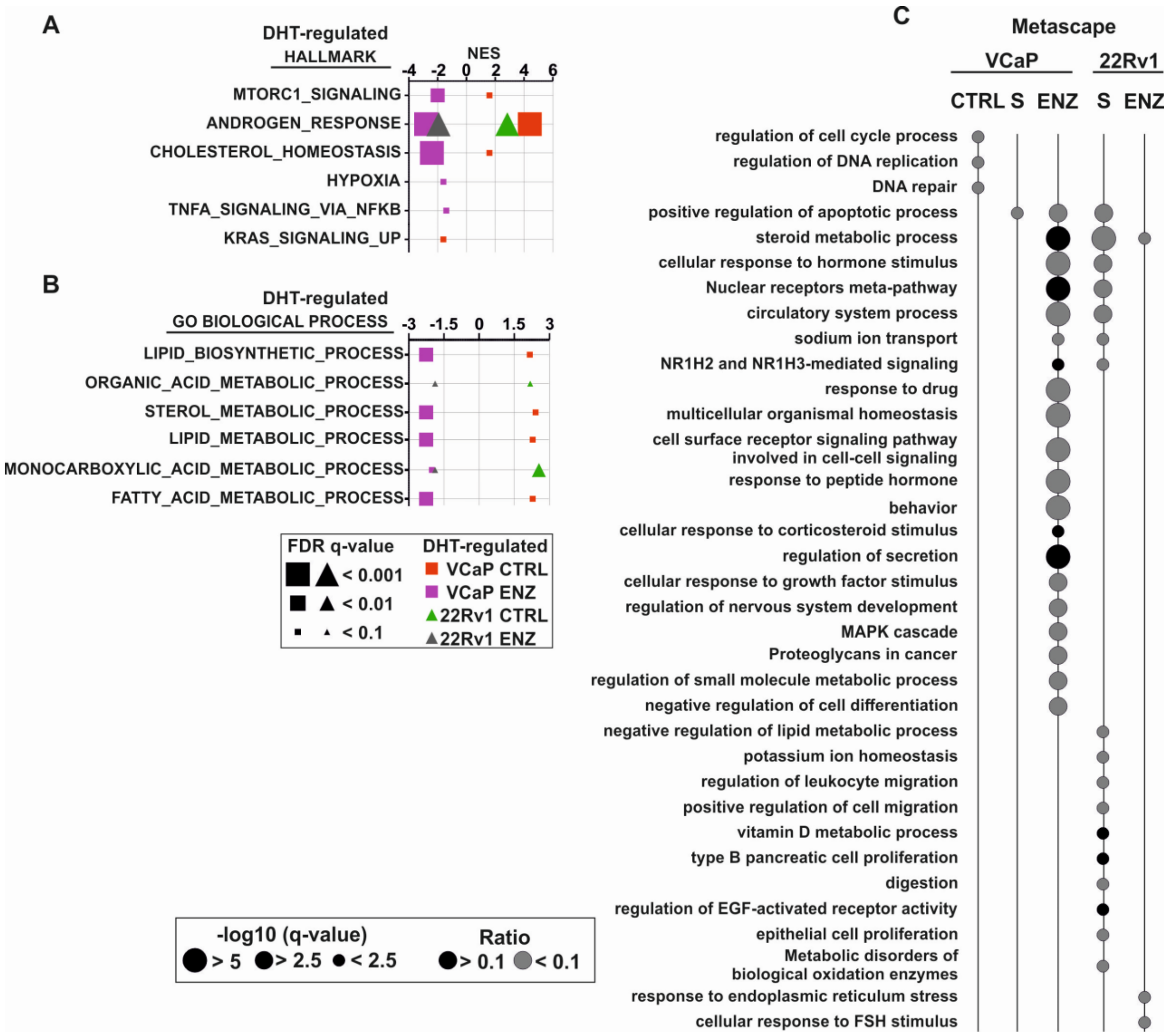

Supplementary Figure S6. Pathway analysis of enzalutamide treated VCaP and 22Rv1 cells. (A-B) Gene set enrichment analysis (GSEA) of DHT-regulated genes from VCaP or 22Rv1 before (VCaP, red square; 22Rv1, green triangle) or after (VCaP, purple square; 22Rv1, grey triangle) ENZ treatment at hallmark (A) or GO biological process (B) pathways. Data shown as normalized enrichment score (NES) with square and triangle size depicting FDR q-value. (C) Metascape pathway analysis of Dex-regulated genes from VCaP and 22Rv1 cells from gene clusters. Circle size depicts -log10 q-value, and black circle indicated over 0.1 and grey circle less than 0.1 gene ratio.

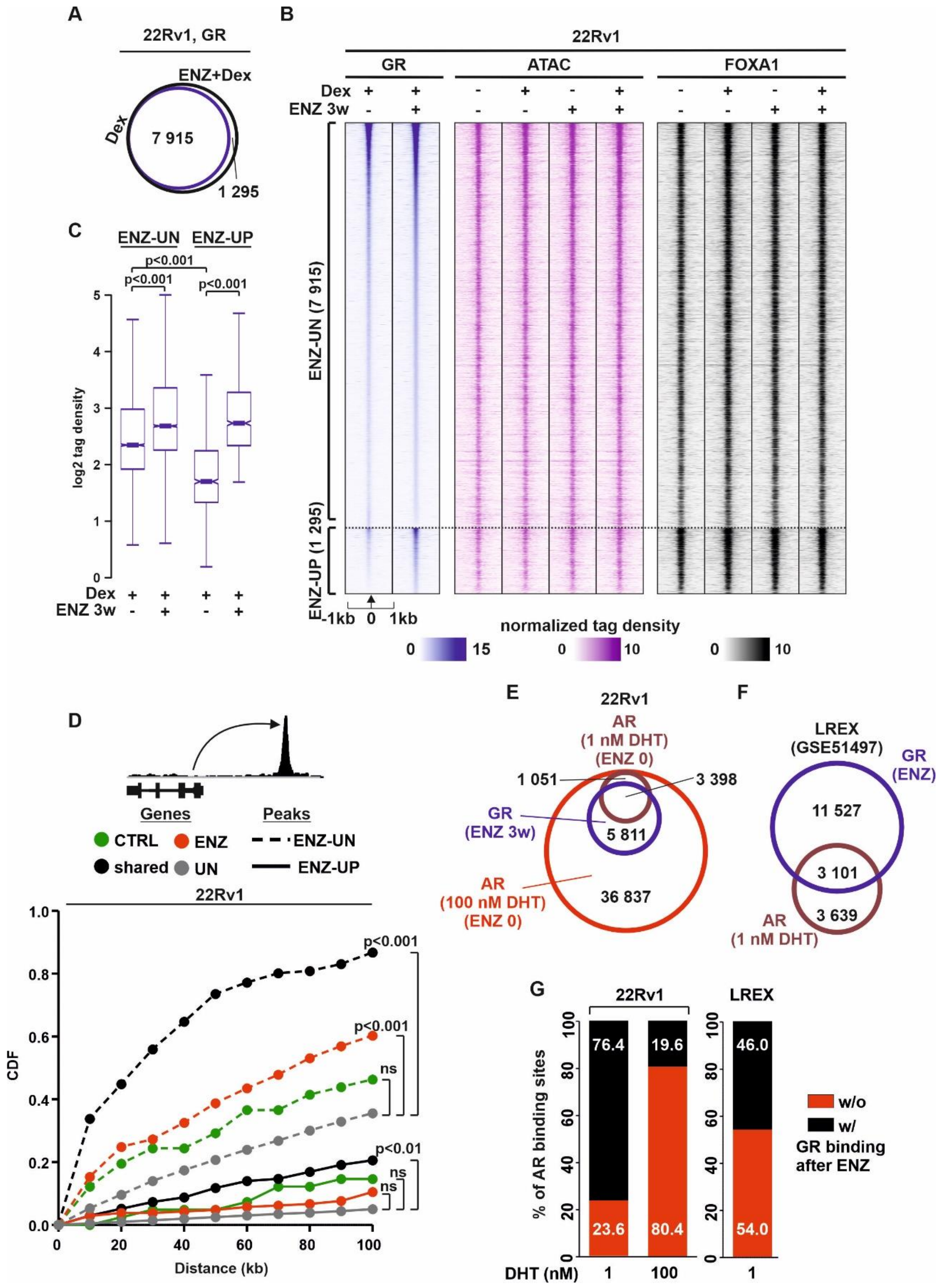

Supplementary Figure S7. Chromatin analysis of GR action in 22Rv1 cells. (A) Venn diagrams of GR chromatin binding in 22Rv1 cells from GR ChIP-seq after Dex (blue circle) or ENZ+Dex (black circle) treatment. (B) GR ChIP-seq, ATAC-seq, and FOXA1 ChIP-seq profiles at ENZ-UN and ENZ-UP sites in 22Rv1 ENZ 0 and ENZ 3w cells. ENZ-UN represents unchanged and ENZ-UP increased GR binding sites. Each heatmap represents ±1 kb around the center of the GR peak. Binding intensity (tags per bp per site) scale is noted below on a linear scale. (C) Box plots represent the normalized log2 tag density of GR ChIP-seq at ENZ-UN and ENZ-UP sites. Statistical significance calculated using One-way ANOVA with Bonferroni *post hoc* test. (D) Association of Dex-regulated genes in 22Rv1 ENZ 0 and ENZ 3w cells to ENZ-UN and ENZ-UP GR binding sites. Different subsets of Dex-regulated genes are color coded, and ENZ-UN depicted as dotted line and ENZ-UP as solid line. Statistical significance calculated with Kolmogorov–Smirnov test. (E) Venn diagrams of GR and AR chromatin binding in 22Rv1 cells from GR ChIP-seq treated with ENZ 3w (blue circle) and AR ChIP-seq treated with 1 nM (magenta circle) or 100 nM (red circle) of DHT. (F) Venn diagrams of GR and AR chromatin binding in LREX cells from GR ChIP-seq treated with ENZ (blue circle) and AR ChIP-seq treated with 1 nM (magenta circle) of DHT. (G) Bar graphs depict percentage overlap of AR binding sites with (black) or without (red) GR binding in 22Rv1 and LREX cells. All heatmaps and box plots are normalized to a total of 10 million reads.

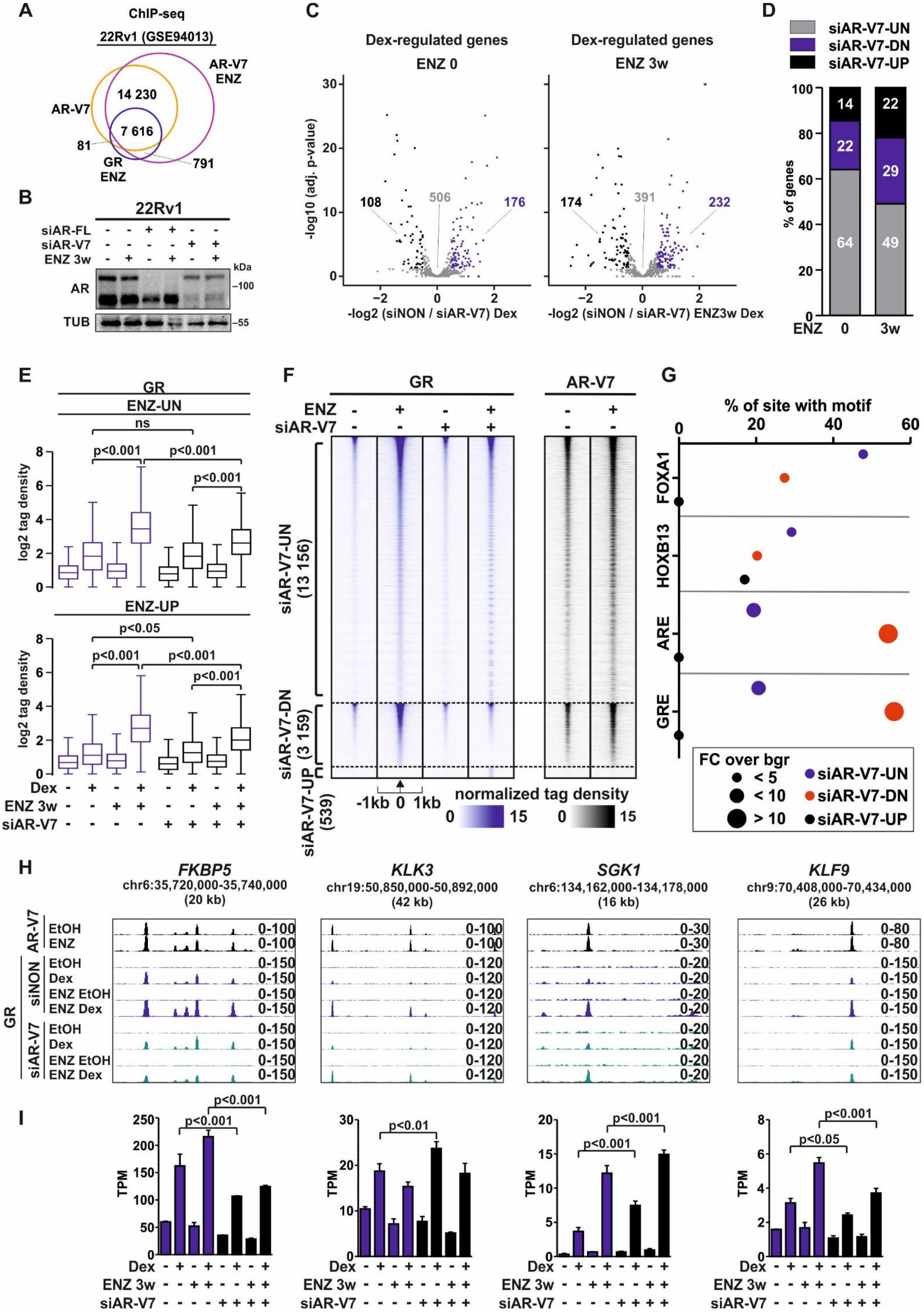
A
ChIP-seq
22Rv1 (GSE94013)
AR-V7
AR-V7 ENZ
GR ENZ
14 230
7 616
81
791
B
22Rv1
siAR-FL
siAR-V7
ENZ 3w
kDa
AR
TUB
-100
-55
C
Dex-regulated genes
ENZ 0
ENZ 3w
-log10 (adj. p-value)
108
506
176
174
391
232
-log2 (siNON / siAR-V7) Dex
-log2 (siNON / siAR-V7) ENZ3w Dex
D
siAR-V7-UN
siAR-V7-DN
siAR-V7-UP
% of genes
14
22
64
22
29
49
ENZ
0
3w
E
GR
ENZ-UN
ns
p<0.001
p<0.001
p<0.001
log2 tag density
ENZ-UP
p<0.05
p<0.001
p<0.001
p<0.001
Dex
ENZ 3w
siAR-V7
F
GR
AR-V7
ENZ
siAR-V7
siAR-V7-UN (13 156)
siAR-V7-DN (3 159)
siAR-V7-UP (539)
-1kb 0 1kb
normalized tag density
0
15
G
% of site with motif
FOXA1
HOXB13
ARE
GRE
FC over bgr
< 5
< 10
> 10
siAR-V7-UN
siAR-V7-DN
siAR-V7-UP
H
FKBP5
chr6:35,720,000-35,740,000
(20 kb)
KLK3
chr19:50,850,000-50,892,000
(42 kb)
SGK1
chr6:134,162,000-134,178,000
(16 kb)
KLF9
chr9:70,408,000-70,434,000
(26 kb)
AR-V7
EtOH
ENZ
GR
siNON
EtOH
Dex
ENZ EtOH
ENZ Dex
siAR-V7
EtOH
Dex
ENZ EtOH
ENZ Dex
0-100
0-150
0-120
0-30
0-20
0-80
I
TPM
p<0.001
p<0.001
p<0.01
p<0.001
p<0.001
p<0.001
p<0.05
Dex
ENZ 3w
siAR-V7

Supplementary Figure S8. The effect of AR-V7 depletion on the transcriptional activity of GR in 22Rv1 cells. (A) Chromatin binding overlap of GR ChIP-seq after ENZ treatment (blue) and AR-V7 ChIP-seq before (yellow) and after (purple) ENZ treatment in 22Rv1 cells. (B) Immunoblotting of AR and TUB protein levels in 22Rv1 ENZ 0 and ENZ 3w cells treated with control or AR-FL or AR-V7 (siAR-V7) specific siRNA. (C) Volcano plot of Dex-regulated genes depicting log2 fold change values of siNON / siAR-V7 (x-axis) and -log10 adjusted p-value (y-axis) from Dex-treated cells (left) or ENZ and Dex-treated cells (right). Number of Dex-regulated genes that are unchanged (grey), decreased (blue), or increased (black) after siAR-V7 treatment (FDR<0.05, log2 fold change±0.5) are indicated in the plot. (D) Bar graph depicting the proportion of Dex-regulated genes that are unchanged (siAR-V7-UN, grey), decreased (siAR-V7-DN, blue), or increased (siAR-V7-UP, black) after siAR-V7 treatment in ENZ 0 (left) or ENZ 3w (right) treated 22Rv1 cells. (E) Box plots represent the normalized log2 tag density of GR ChIP-seq at ENZ-UN and ENZ-UP sites with or without AR-V7 depletion in 22Rv1 ENZ 0 and ENZ 3w cells. (F) GR and AR-V7 ChIP-seq profiles at siAR-V7-UN, siAR-V7-DN and siAR-V7-UP sites in 22Rv1 ENZ 0 and ENZ 3w cells with or without AR-V7 depletion. siAR-V7-UN represents unchanged, siAR-V7-DN decreased and siAR-V7-UP increased GR binding sites. Each heatmap represents ±1 kb around the center of the GR peak. Binding intensity (tags per bp per site) scale is noted below on a linear scale. (G) *De novo* motif enrichment of FOXA1, HOXB13, ARE and GRE motifs at the siAR-V7-UN (blue), siAR-V7-DN (orange) and siAR-V7-UP (black) sites in 22Rv1 cells. X-axis represents percentage of sites with motifs, and circle size depicts fold change (FC) over the background. (H) Genome browser tracks of *FKBP5*, *KLK3* (PSA), *SGK1* and *KLF9* loci depicting AR-V7 and GR ChIP-seq in 22Rv1 ENZ 0 and ENZ 3w cells treated with siNON or siAR-V7. (I) Bar graphs depict GR target gene analysis of *FKBP5*, *KLK3*, *SGK1*, *KLF9* in 22Rv1 ENZ 0 and ENZ 3w cells treated with siNON or siAR-V7 in the presence or absence of Dex. Bar graphs depict TPM values of *FKBP5*, *KLK3*, *SGK1*, *KLF9* in 22Rv1 ENZ 0 and ENZ 3w cells treated with siNON or siAR-V7 in the presence or absence of Dex from RNA-seq data. Bars represent mean ±SD, n=3. All genome browser tracks, and box plots are normalized to a total of 10 million reads. Statistical significance calculated with One-way ANOVA with Bonferroni *post hoc* test (3 conditions).

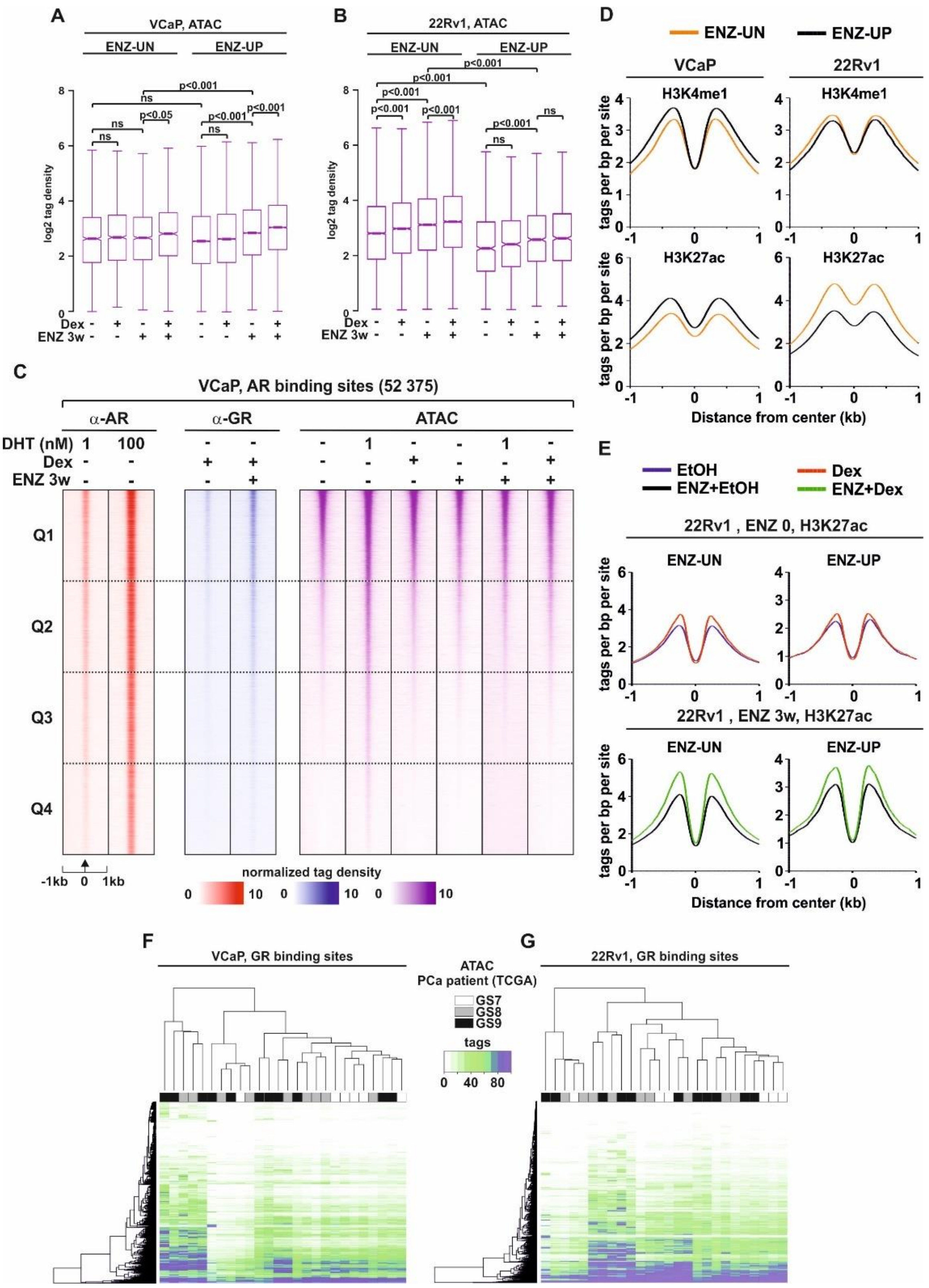

Supplementary Figure S9. Chromatin accessibility and histone modification enrichment at GR binding sites. (A-B) Box plots represent the normalized log2 tag density of ATAC-seq at ENZ-UN and ENZ-UP sites in (A) VCaP and (B) 22Rv1 cells. Statistical significance calculated using One-way ANOVA with Bonferroni *post hoc* test. (C) AR ChIP-seq, GR ChIP-seq and ATAC-seq profiles at AR binding sites in VCaP cells. AR binding sites were sorted based on pre-accessibility to quartiles with Q1 the most and Q4 the least pre-accessible. Each heatmap represents ±1 kb around the center of the AR peak. Binding intensity (tags per bp per site) scale is noted below on a linear scale. (D) Aggregate plots represent the binding intensity (tags per bp per site) of H3K4me1 (upper) and H3K27ac (lower) ChIP-seq at ENZ-UN and ENZ-UP sites in untreated VCaP and 22Rv1 cells. (E) Aggregate plots represent the binding intensity (tags per bp per site) of H3K27ac ChIP-seq at ENZ-UN and ENZ-UP sites in 22Rv1 ENZ 0 (upper) and ENZ 3w (lower) cells. Each aggregate plot represents ±1 kb around the center of the GR peak. (F-G) Heatmap of ATAC-seq from PCa patients at GR binding sites in (F) VCaP and (G) 22Rv1 cells. Columns represent different patients with color code depicting patient Gleason score (GS). Rows represent individual GR binding sites. Binding intensity scale is shown on a linear scale. All heatmaps, aggregate and box plots are normalized to a total of 10 million reads.

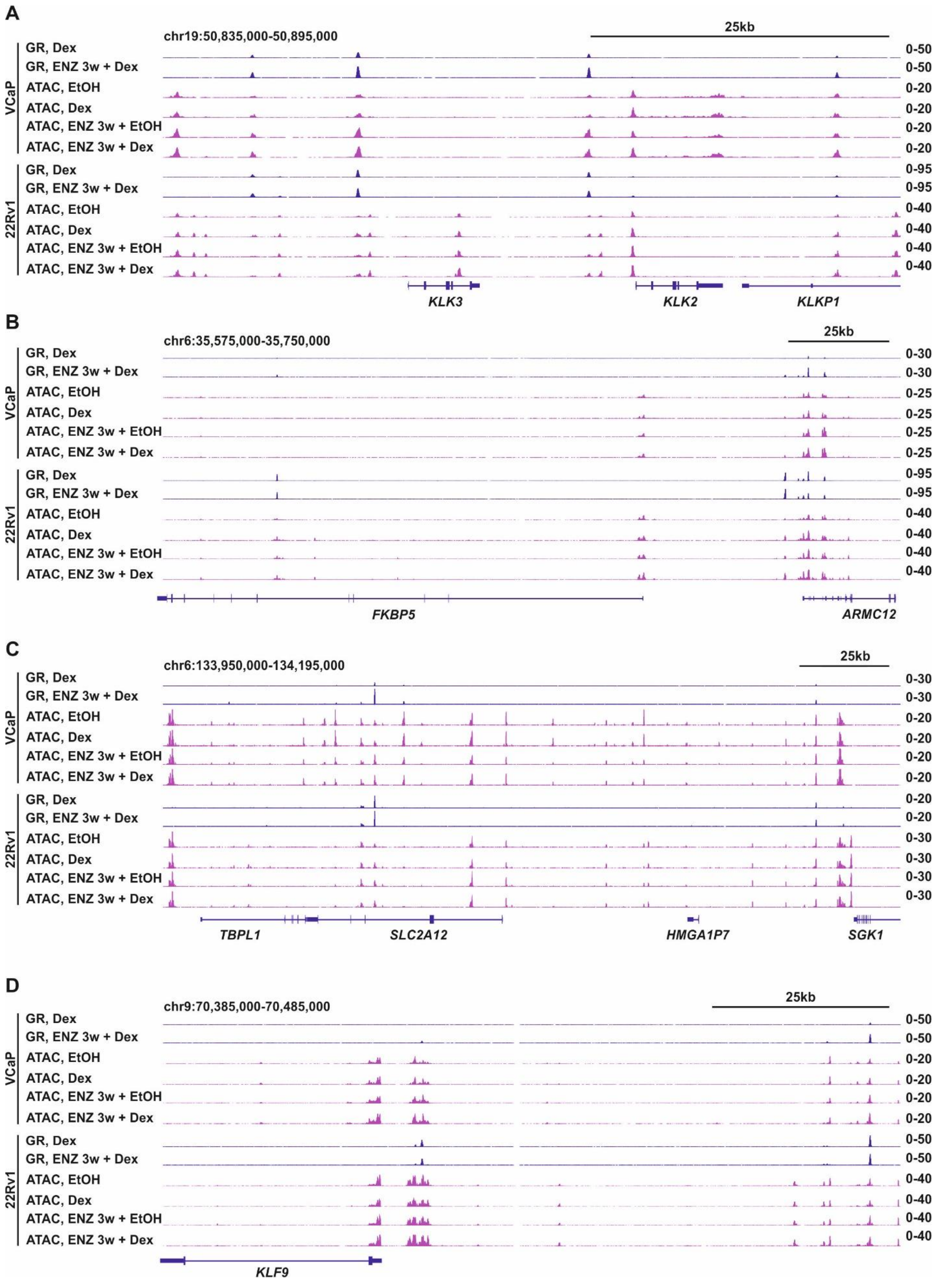

Supplementary Figure S10. Example genome browser tracks. Genome browser tracks of (A) *KLK3*, (B) *FKBP5*, (C) *SGK1*, and (D) *KLF9* loci depicting GR ChIP-seq and ATAC-seq in VCaP and 22Rv1 ENZ 0 and ENZ 3w cells treated with or without Dex. All genome browser tracks are normalized to a total of 10 million reads.

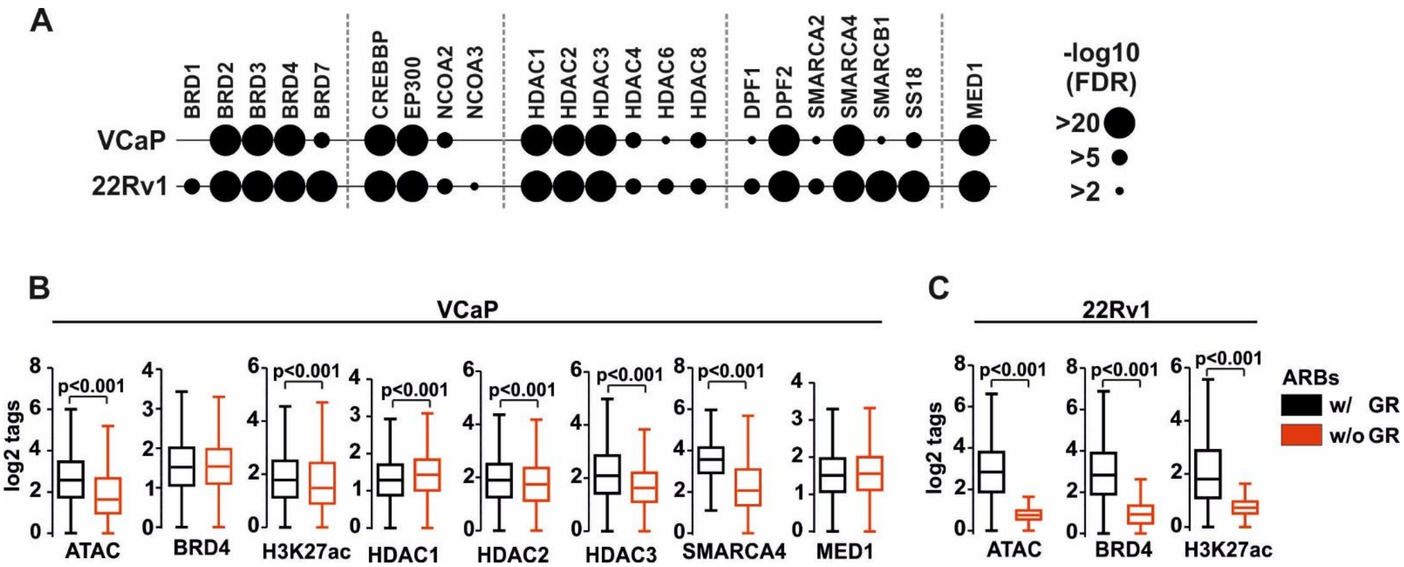

Supplementary Figure S11. Enrichment of coregulators at AR binding sites with or without GR occupancy. (A) TcoF gene set enrichment of Dex-regulated genes in VCaP and 22Rv1 cells. Circle size depicts -log10 FDR value. (B) Box plots represent the normalized log2 tag density of ATAC-seq, BRD4 ChIP-seq, H3K27ac ChIP-seq, HDAC1 ChIP-seq, HDAC2 ChIP-seq, HDAC3 ChIP-seq, SMARCA4 ChIP-seq, and MED1 ChIP-seq at AR binding sites with (w/; black) or without (w/o; red) GR occupancy in VCaP cells. (C) Box plots represent the normalized log2 tag density of ATAC-seq, BRD4 ChIP-seq, and H3K27ac ChIP-seq at AR binding sites with (w/; black) or without (w/o; red) GR occupancy in 22Rv1 cells. All box plots are normalized to a total of 10 million reads. Statistical significance calculated with unpaired t-test.

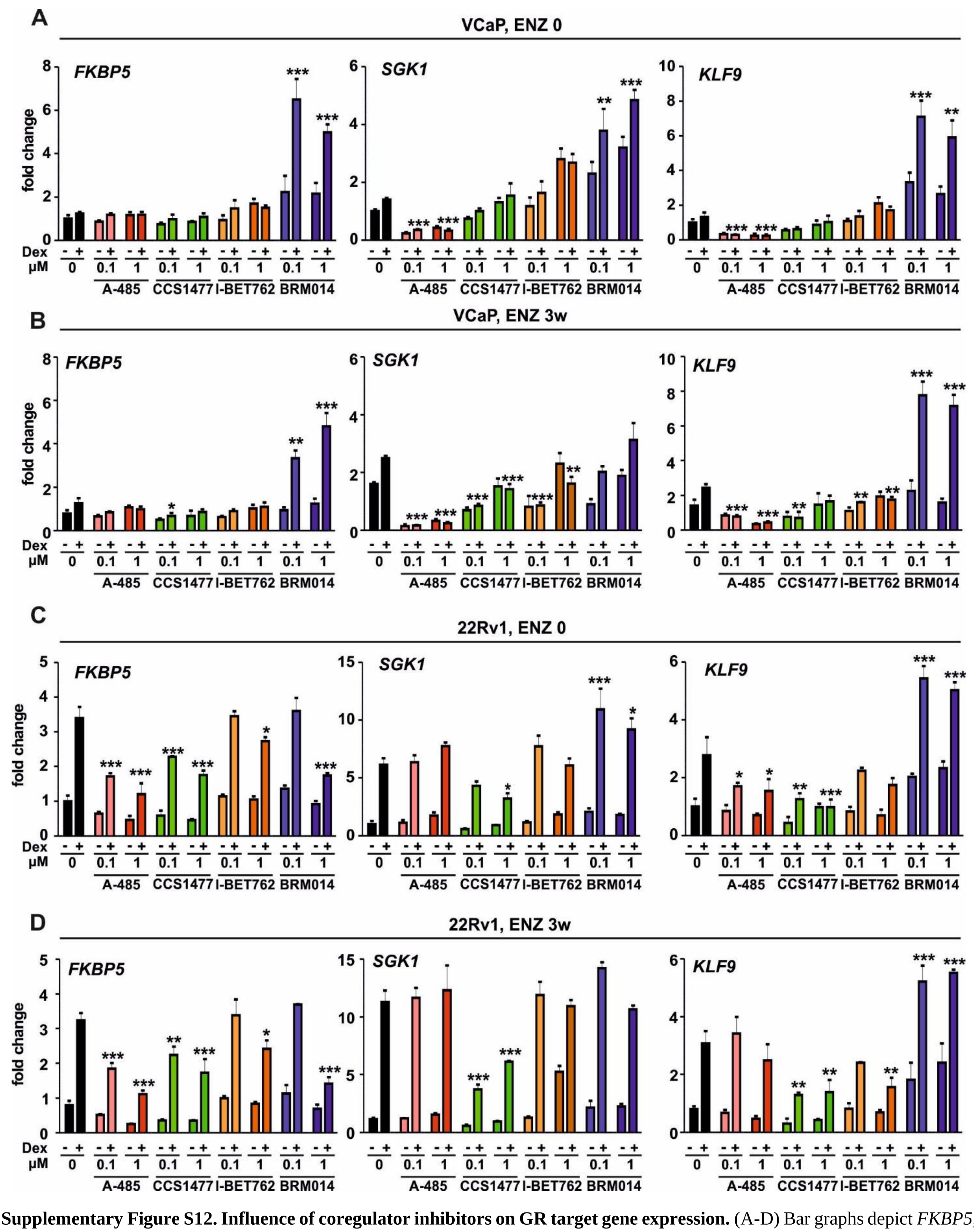

Supplementary Figure S12. Influence of coregulator inhibitors on GR target gene expression. (A-D) Bar graphs depict *FKBP5*, *SGK1*, and *KLF9* gene expression analysis in VCaP ENZ 0 (A), VCaP ENZ 3w (B), 22Rv1 ENZ 0 (C), and 22Rv1 ENZ 3w (D) cells treated with or without Dex in the presence or absence of 0.1 or 1 µM of indicated inhibitor. Bars represent mean ±SD, n=3. Statistical significance calculated using One-way ANOVA with Bonferroni *post hoc* test. *, $p<0.05$; **. $p<0.01$; ***, $p<0.001$.

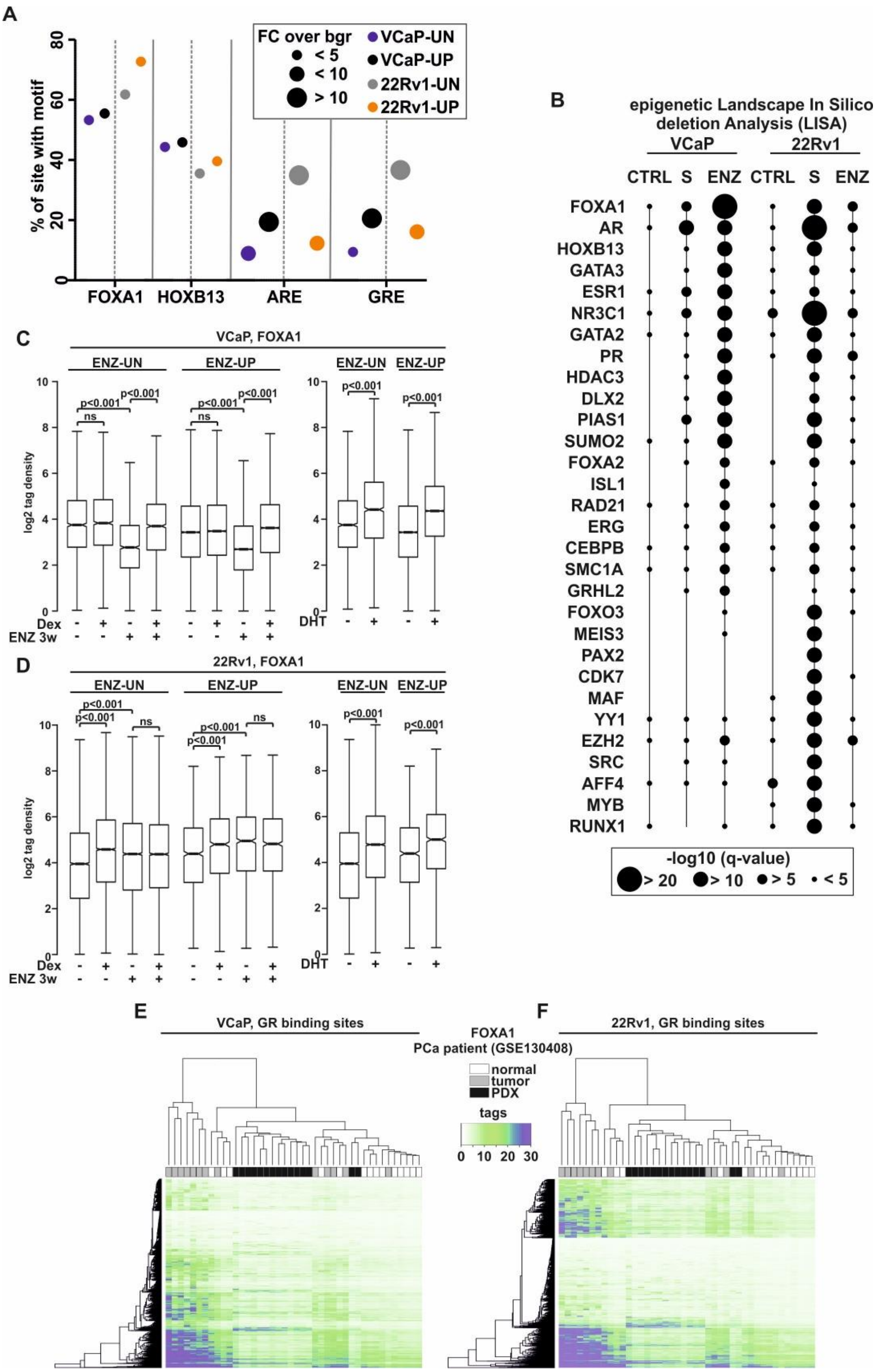

Supplementary Figure S13. Analysis of FOXA1 enrichment at GR binding sites. (A) *De novo* motif enrichment of FOXA1, HOXB13, ARE, and GRE motifs at the ENZ-UN (VCaP, blue; 22Rv1 grey) and ENZ-UP (VCaP, black; 22Rv1, orange) sites in VCaP and 22Rv1 cells. Y-axis represents percentage of sites with motifs, and circle size depicts fold change (FC) over the background. (B) Epigenetic landscape in silico deletion analysis (LISA) of Dex-regulated genes from VCaP and 22Rv1 cells from indicated gene clusters. Circle size depicts -log10 q-value. (C-D) Box plots represent the normalized log2 tag density of FOXA1 ChIP-seq at ENZ-UN and ENZ-UP sites in (C) VCaP and (D) 22Rv1 cells. Statistical significance calculated using One-way ANOVA with Bonferroni *post hoc* test. (E-F) Heatmap of FOXA1 ChIP-seq from PCa patients at GR binding sites in (E) VCaP and (F) 22Rv1 cells. Columns represent different patients with color code depicting sample type. Rows represent individual GR binding sites. Binding intensity scale is shown on a linear scale. All heatmaps and box plots are normalized to a total of 10 million reads.

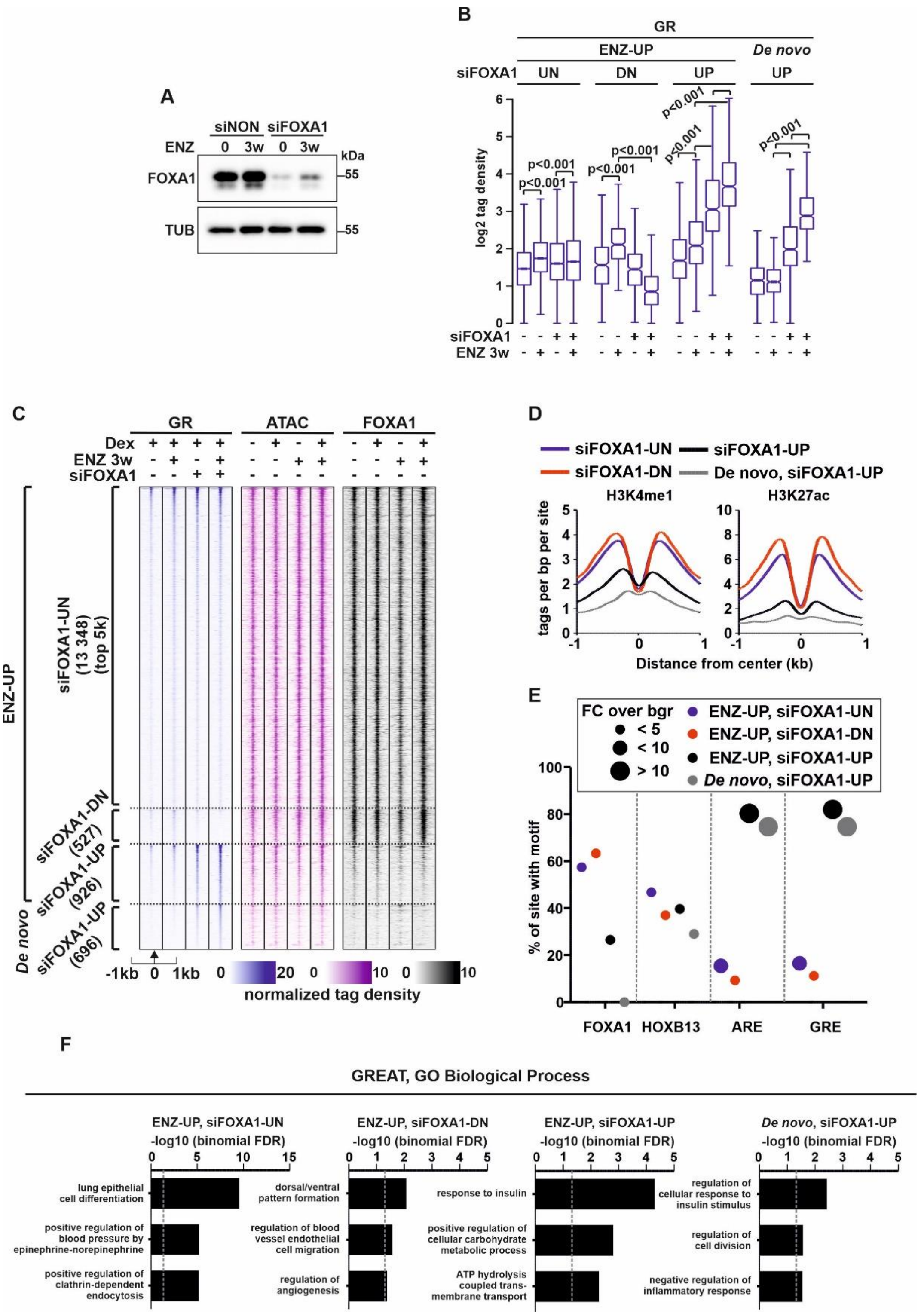

Supplementary Figure S14. Depletion of FOXA1 increases GR chromatin binding. (A) Immunoblotting of FOXA1 and TUB protein levels in VCaP ENZ 0 and ENZ 3w cells treated with siNON or siFOXA1. (B) Box plots represent the normalized log2 tag density of GR ChIP-seq at ENZ-UP siFOXA1-UN, ENZ-UP siFOXA1-DN, ENZ-UP siFOXA1-UP, and *de novo* siFOXA1-UP sites in VCaP cells. Statistical significance calculated using One-way ANOVA with Bonferroni *post hoc* test. (C) GR ChIP-seq, ATAC-seq, and FOXA1 ChIP-seq profiles at ENZ-UP siFOXA1-UN, ENZ-UP siFOXA1-DN, ENZ-UP siFOXA1-UP, and *de novo* siFOXA1-UP sites in VCaP cells. Each heatmap represents ±1 kb around the center of the GR peak. Binding intensity (tags per bp per site) scale is noted below on a linear scale. (D) Aggregate plots represent the binding intensity (tags per bp per site) of H3K4me1 (left) and H3K27ac (right) ChIP-seq at ENZ-UP siFOXA1-UN, ENZ-UP siFOXA1-DN, ENZ-UP siFOXA1-UP, and *de novo* siFOXA1-UP sites in VCaP cells. (E) *De novo* motif enrichment of FOXA1, HOXB13, ARE, and GRE motifs at the ENZ-UP siFOXA1-UN (blue), ENZ-UP siFOXA1-DN (red), ENZ-UP siFOXA1-UP (black), and *de novo* siFOXA1-UP (grey) sites in VCaP cells. Y-axis represents percentage of sites with motifs, and circle size depicts fold change (FC) over the background. (F) GREAT enrichment analysis at GO Biological Process pathways for of ENZ-UP siFOXA1-UN, ENZ-UP siFOXA1-DN, ENZ-UP siFOXA1-UP, and *de novo* siFOXA1-UP sites in VCaP cells. Each bar depicts -log10 binomial FDR values, and grey dashed line depicts binomial FDR value of 0.05. All heatmaps, aggregate and box plots are normalized to a total of 10 million reads.

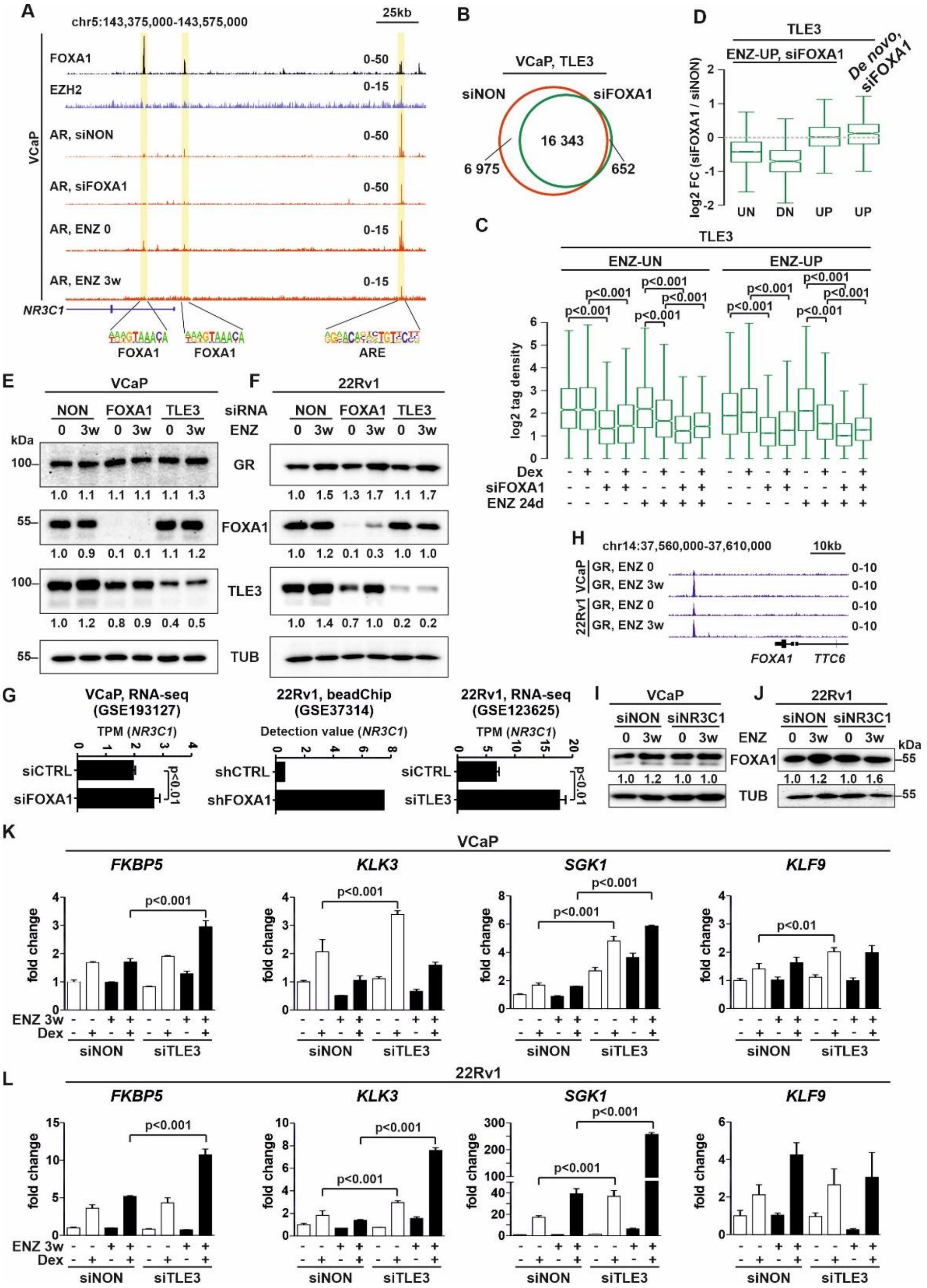

Supplementary Figure S15. Regulation of NR3C1 by FOXA1. (A) Genome browser tracks of *NR3C1* loci depicting FOXA1 ChIP-seq, EZH2 ChIP-seq, and AR ChIP-seq in VCaP cells. AR ChIP-seq is shown in cells treated with siNON or siFOXA1, and in ENZ 0 and ENZ 3w cells. Location of FOXA1 and ARE binding motifs are indicated at the bottom of the tracks. (B) Venn diagrams of TLE3 chromatin binding in VCaP cells treated with siNON (red circle) or siFOXA1 (green circle). (C) Box plots represent the normalized log2 tag density of TLE3 ChIP-seq at ENZ-UN and ENZ-UP sites in VCaP cells treated with or without siFOXA1. (D) Log2 fold change (FC) of TLE3 ChIP-seq tags treated with siFOXA1 versus treated with siNON at ENZ-UP siFOXA1-UN, ENZ-UP siFOXA1-DN, ENZ-UP siFOXA1-UP, and *de novo* siFOXA1-UP sites in VCaP cells. (E-F) Immunoblotting of GR, FOXA1, TLE3, and TUB protein levels in VCaP (E) and 22Rv1 (F) ENZ 0 and ENZ 3w cells treated with siNON or siFOXA1 or siTLE3. Quantification of GR, FOXA1, and TLE3 levels to TUB is shown below the immunoblot. (G) Transcript levels of NR3C1 using indicated method in VCaP cells after siFOXA1 (left), in 22Rv1 cells after shFOXA1 (middle), and in 22Rv1 cells after siTLE3 (right). (H) Genome browser tracks of *FOXA1* loci depicting GR ChIP-seq in VCaP and 22Rv1 ENZ 0 and ENZ 3w cells. (I-J) Immunoblotting of FOXA1 and TUB protein levels in (I) VCaP or (J) 22Rv1 ENZ 0 and ENZ 3w cells treated with siNON or siNR3C1. Quantification of FOXA1 levels to TUB is shown below the immunoblot. (K-L) Bar graphs depict GR target gene analysis of *FKBP5*, *KLK3*, *SGK1*, *KLF9* in VCaP (K) and 22Rv1 (L) ENZ 0 and ENZ 3w cells treated with siNON or siTLE3 in the presence or absence of Dex. Bars represent mean ±SD, n=3. All genome browser tracks, and box plots are normalized to a total of 10 million reads. Statistical significance calculated with unpaired t-test (2 conditions) or using One-way ANOVA with Bonferroni *post hoc* test (3 conditions).

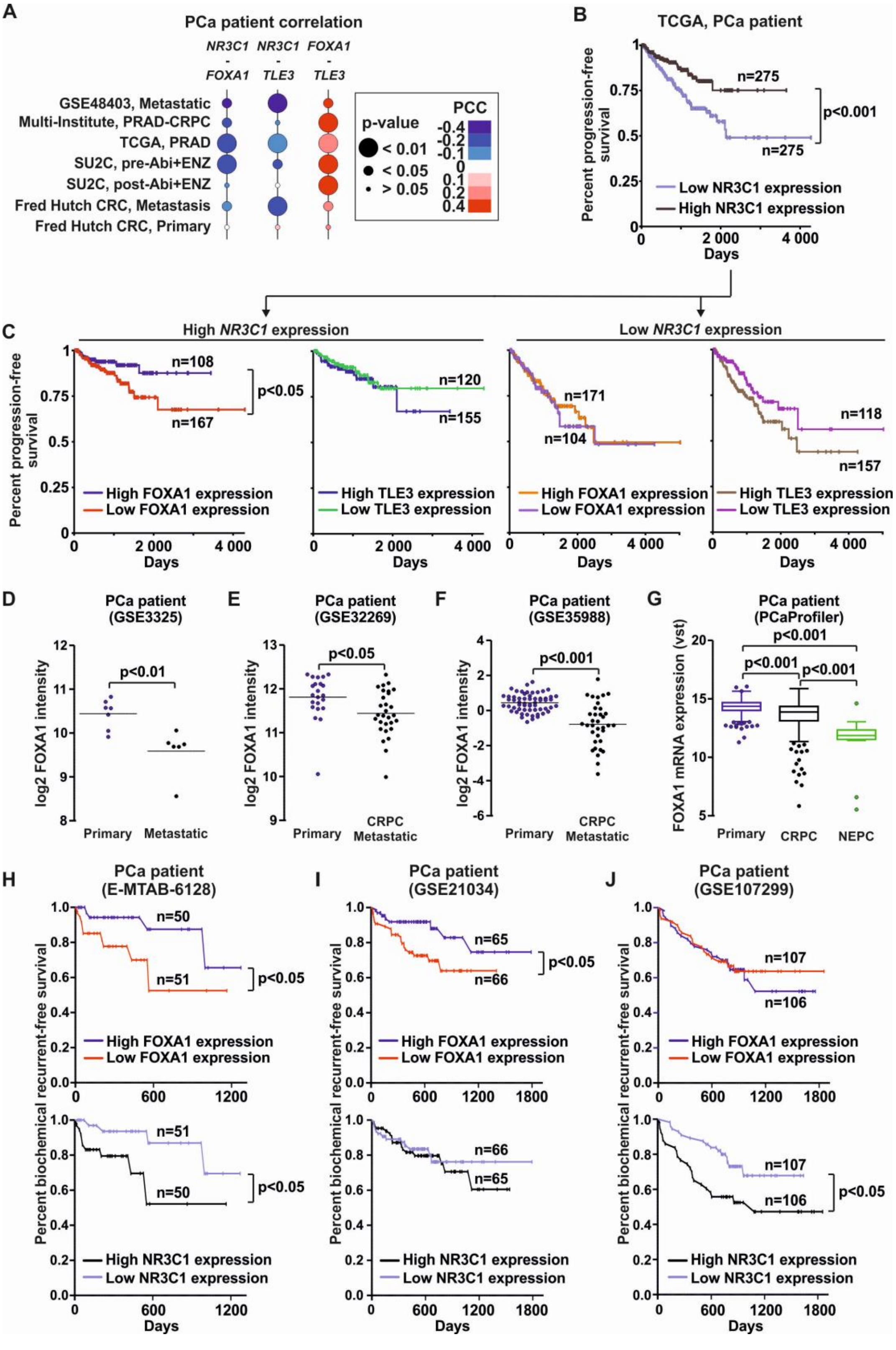

Supplementary Figure S16. Prostate cancer patient dataset analysis. (A) Pearson correlation coefficiency (PCC) of *NR3C1*, *FOXA1*, and *TLE3* transcript levels at indicated PCa patient datasets. Circle color represents PCC value with blue indicating negative and red positive correlation. Circle size depicts p-value. (B) Progression-free survival of TCGA PCa patients divided into low (light blue) and high (black) *NR3C1* expression. (C) Progression-free survival of TCGA PCa patients with high (left) or low (right) *NR3C1* expression divided into low and high *FOXA1* or low and high *TLE3* expression. Color coding: high *NR3C1* high *FOXA1*, blue; high *NR3C1* low *FOXA1*, red; high *NR3C1* high *TLE3*, dark blue; high *NR3C1* low *TLE3*, green; low *NR3C1* high *FOXA1*, orange; low *NR3C1* low *FOXA1*, purple; low *NR3C1* high *TLE3*, brown; low *NR3C1* low *TLE3*, violet. (D-G) Transcript levels of *FOXA1* from (D) primary and metastatic PCa patients, (E-F) primary and CRPC metastatic PCa patients, (G) primary, CRPC and neuroendocrine (NEPC) PCa patients. (H-J) Biochemical recurrent-free survival of three PCa patient datasets divided into low (red) and high (blue) *FOXA1* expression (upper), or low (light blue) and high (black) *NR3C1* expression (lower). Data accession number is indicated for each dataset. Statistical significance is calculated with log-rank test in the survival graphs, and with unpaired t-test (2 conditions) or One-way ANOVA with Bonferroni *post hoc* test (3 conditions) in the box plots.

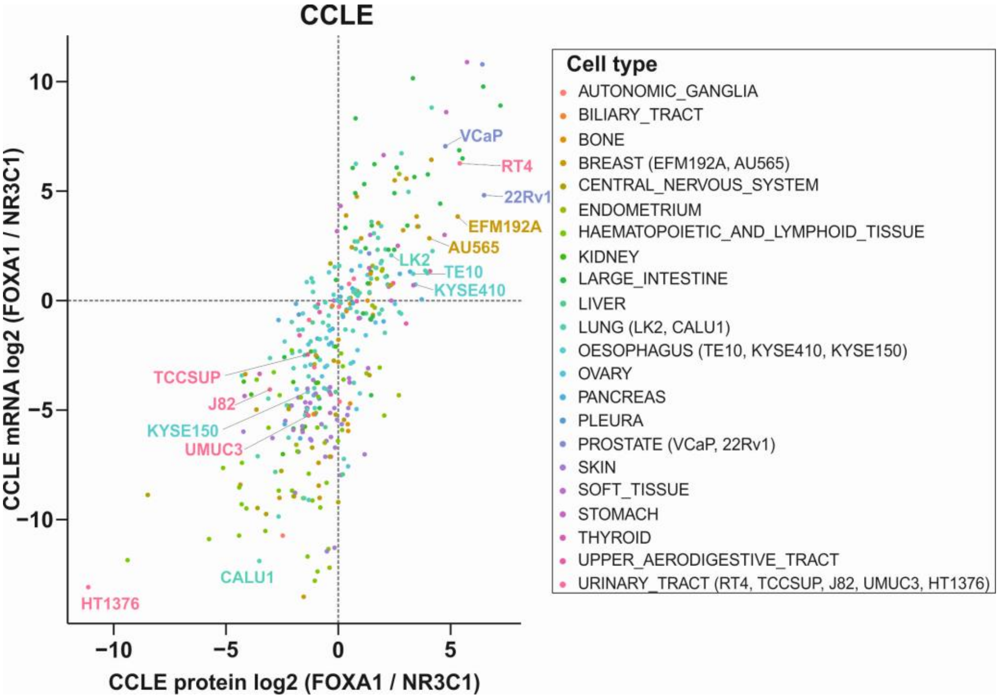

Supplementary Figure S17. Analysis of *FOXA1* and *NR3C1* expression from cancer cell line encyclopedia (CCLE) dataset. Log2 fold change of *FOXA1* versus *NR3C1* transcript (y-axis) and protein (x-axis) expression in CCLE cell types. Cell types are color coded with anatomical location. Specific cell lines are highlighted in the graph and in the legend.

Supplementary Table S1. Custom siRNA sequences and RT-qPCR primers.

siAR-FL (exon 7)	Sense: 5'-UCAAGGAACUCGAUCGUAU-3'<br>Antisense: 5'-AUACGAUCGAGUUCCUUGA-3'
siAR-V7 (cryptic exon)	Sense: 5'-GUAGUUGUGAGUAUCAUGA-3'<br>Antisense: 5'-UCAUGAUACUCACAACUAC-3'
FKBP5	Forward: 5'-AAAAGGCCAAGGAGCACAAC-3'<br>Reverse: 5'-TTGAGGAGGGGCCGAGTTC-3'
KLK3 (PSA)	Forward: 5'-GGCAGGTGCTTGTGGCCTCTC-3'<br>Reverse: 5'-CACCCGAGCAGGTGCTTTTGC-3'
SGK1	Forward: 5'-ATGCCAACCCTTCTCCTCC-3'<br>Reverse: 5'-TCAACAGAACATTCCGCTCC-3'
KLF9	Forward: 5'-TACAGTGGCTGTGGGAAAGT-3'<br>Reverse: 5'-AGCGGGAGAACTTTTTAAGG-3'
NR3C1 (GR)	Forward: 5'-GAACTGGCAGCGGTTTTATC-3'<br>Reverse: 5'-TGGTATCTGATTGGTGATGATTTC-3'
RPL13A	Forward: 5'-ACGACAAGAAAAAGCGGATGG-3'<br>Reverse: 5'-AGGGCAACAATGGAGGAAGG-3'

Supplementary Table S2. RNA-seq data.

Supplementary Table S3. Pathway enrichment analyses.

Supplementary Table S4. Public datasets utilized in the study.

Supplementary Table S5. ChIP-seq peaks.

Supplementary Table S6. Motif enrichment analyses.
